# The effect of the COVID-19 pandemic on mental health calls for police service

**DOI:** 10.1186/s40163-021-00157-6

**Published:** 2021-10-11

**Authors:** Jacek Koziarski

**Affiliations:** Department of Sociology, University of Western Ontario, 1151 Richmond Street, London, ON N6A 3K7 Canada

**Keywords:** Police, Mental Health, COVID-19, Bayesian Structural Time Series

## Abstract

Drawing upon seven years of police calls for service data (2014–2020), this study examined the effect of the COVID-19 pandemic on calls involving persons with perceived mental illness (PwPMI) using a Bayesian Structural Time Series. The findings revealed that PwPMI calls did not increase immediately after the beginning of the pandemic in March 2020. Instead, a sustained increase in PwPMI calls was identified in August 2020 that later became statistically significant in October 2020. Ultimately, the analysis revealed a 22% increase in PwPMI calls during the COVID-19 pandemic than would have been expected had the pandemic not taken place. The delayed effect of the pandemic on such calls points to a need for policymakers to prioritize widely accessible mental health care that can be deployed early during public health emergencies thus potentially mitigating or eliminating the need for increased police intervention, as was the case here.

**Supplementary Information:**

The online version contains supplementary material available at 10.1186/s40163-021-00157-6.

## Introduction

In March of 2020, the World Health Organization declared Coronavirus Disease 2019 (COVID-19) as a pandemic (World Health Organization, 2020). Such major public health emergencies have the ability to foster feelings of fear, uncertainty, and loneliness due to increased risk of morbidity and death; public health measures meant to curb virus transmission, such as physical distancing, lockdowns, or quarantines; as well as possible or experienced unemployment, among other reasons (Fitzpatrick et al., 2020; Moreno et al., 2020; Rajkumar, 2020; Vigo et al., 2020; Vindegaard & Benros, 2020; Xiong et al., 2020). These factors, either alone or in combination, can in turn translate into higher levels of psychological distress (Pfefferbaum & North, 2020). In fact, since the onset of the COVID-19 pandemic, a broad body of literature has documented the detrimental effects the pandemic has had on mental health across the globe. More specifically, numerous studies from Canada (e.g., Jenkins et al., 2021; Zajacova et al., 2020), the United States (Holman et al., 2020), the United Kingdom (e.g., Daly et al., 2020; Proto & Quintana-Domeque, 2021), and elsewhere (e.g., Arendt et al., 2020; Rajkumar, 2020; Vindegaard & Benros, 2020; Wang et al., 2020; Xiong et al., 2020) have reported either deteriorations in self-reported mental health or heightened levels of specific symptoms—such as post-traumatic stress symptoms (PTSS), stress, anxiety, and/or depression—among their respective general populations during the COVID-19 pandemic. Other studies that have examined the effects of specific public health measures on mental health have revealed similar findings. Rossi et al. (2020), for instance, identified high rates of PTSS, depression, anxiety, and stress among the Italian general population during lockdown; whereas Wang et al. (2021) found similarly high rates of depression, anxiety, and stress among individuals subject to quarantine measures in China.

Additional lines of inquiry have also examined the pandemic’s mental health effects on specific sub-populations. Individuals with pre-existing mental health disorders, for instance, have reported increases in their respective symptoms (Vindegaard & Benros, 2020; Xiong et al., 2020), which have likely been additionally exacerbated for some due to disruption of in-person psychiatric care to accommodate public health measures (Moreno et al., 2020; Vigo et al., 2020). Healthcare workers on the frontlines have similarly reported heightened levels of stress, anxiety, and depression during the COVID-19 pandemic due to factors such as immediate exposure to the virus and death, concerns over infection of self and family members, lack of personal protective equipment, overwork, and burnout (Kock et al., 2021; Moitra et al., 2021; Mosheva et al., 2021). Emerging research has also identified that individuals who have recovered or are in-recovery after COVID-19 infection may experience depression or PTSS (Khademi et al., 2021; Vindegaard & Benros, 2020).

Although evidence on the mental health effects of the COVID-19 pandemic is continuously building, research examining the effects of the COVID-19 pandemic on calls for police service involving persons with perceived mental illness (PwPMI) is scant.^1^ In fact, to-date, research examining the impacts of the pandemic on police-related activity has almost exclusively focused on crime (see e.g., Andresen & Hodgkinson, 2020; Ashby, 2020; Buil-Gil et al., 2020; Campedelli et al., 2020; Estévez-Soto, 2021; Felson et al., 2020; Gerell et al., 2020; Hodgkinson & Andresen, 2020; Langton et al., 2021; Payne et al., 2021; Piquero et al., 2021). While this area of inquiry is certainly important, crime only comprises approximately 20–30% of all police calls for service (Wuschke et al., 2017). As such, the effect of the COVID-19 pandemic on the remaining 70–80% of police activity—which includes PwPMI calls—remains relatively unexplored. Further, even though the police have long been the first responders to PwPMI (Bittner, 1967), renewed debates around the role of the police in these calls warrants an investigation as to whether this role becomes more pronounced during a pandemic.

To-date, the only study to explore this area found that mental health-related calls^2^ in Detroit, Michigan decreased early in the COVID-19 pandemic (Lersch, 2020). This particular study, however, only drew upon call data through April 27, 2020. This is arguably *too* early in the pandemic to detect any significant changes in PwPMI calls, which may have increased as mental health deteriorated and in-person psychiatric care was disrupted in subsequent weeks and months. Further, Lersch’s (2020) study is also largely descriptive in nature, meaning it is not optimal for detecting the impact of an intervention. Additional research with a more robust methodological approach is therefore warranted. In light of these existing limitations, the purpose of this study is to conduct a more rigorous examination into the effect of the COVID-19 pandemic on PwPMI calls for police service with a longer time-series that extends to the end of 2020.

## Data and methods

Data for this study were drawn from the computer-aided dispatch system of the Barrie Police Service in Barrie, Ontario, Canada and included all calls for service from January 1, 2014 through December 31, 2020 where the final disposition was ‘Mental Health’ (*N* = 3977). For the purpose of analysis, these data were aggregated at the weekly level (see Table 1). The first and last week in the series were dropped as they did not contain seven days, thus generating 364 total weeks for analysis.

**Table 1:** Descriptive Statistics, PwPMI Calls Per Week, 2014–2020

Year	Min.	1st Quartile	Median	Mean	SD	3rd Quartile	Max.	Total
2014	2	7	11	10.7	4.2	14	22	568
2015	4	8	12	11.9	4.4	15	20	628
2016	0	6	8	8	3.1	11	14	429
2017	1	7	10	9.8	4.3	12	22	520
2018	3	7	9	9.7	3.5	12	18	515
2019	4	9	11	11.4	3.3	13	19	602
2020	4	10	13	13.5	4.7	18	22	715
2014–2020	0	8	11	10.9	4.2	14	22	3977

To analyze the effect of the COVID-19 pandemic on PwPMI calls, a Bayesian Structural Time Series (BSTS) model was estimated. BSTS models are quasi-experimental in nature in that they measure the effect of an intervention by predicting a counterfactual time-series that would have occurred had the intervention not taken place (Brodersen et al., 2015). This counterfactual is predicted by drawing upon: (1) the prior and posterior behaviour of the time-series in question, and (2) contemporaneous covariates that were not affected by the intervention (Brodersen et al., 2015). Unlike crime, which is widely understood to fluctuate based on temperature-related mechanisms (e.g., Andresen & Malleson, 2013; Linning et al., 2017), little is currently known as to what (if anything) is temporally associated with PwPMI calls.^3^ As a result, the current BSTS modelling is univariate in nature. That is, the model was estimated solely using the prior and posterior behaviour of the time-series. However, with 364 weeks in the series, an adequate BSTS model can be inferred in spite of a lack of theoretically—or empirically—informed covariates. Further, a seasonal term was included in the model to account for weekly fluctuations in PwPMI calls. The intervention was set to the week in which the World Health Organization declared COVID-19 as a pandemic (week of March 9, 2020), resulting in 323 pre-intervention weeks and 41 post-intervention weeks. Ten thousand Markov Chain Monte Carlo samples were drawn to obtain more robust inferences.

## Results

**Fig. 1:**
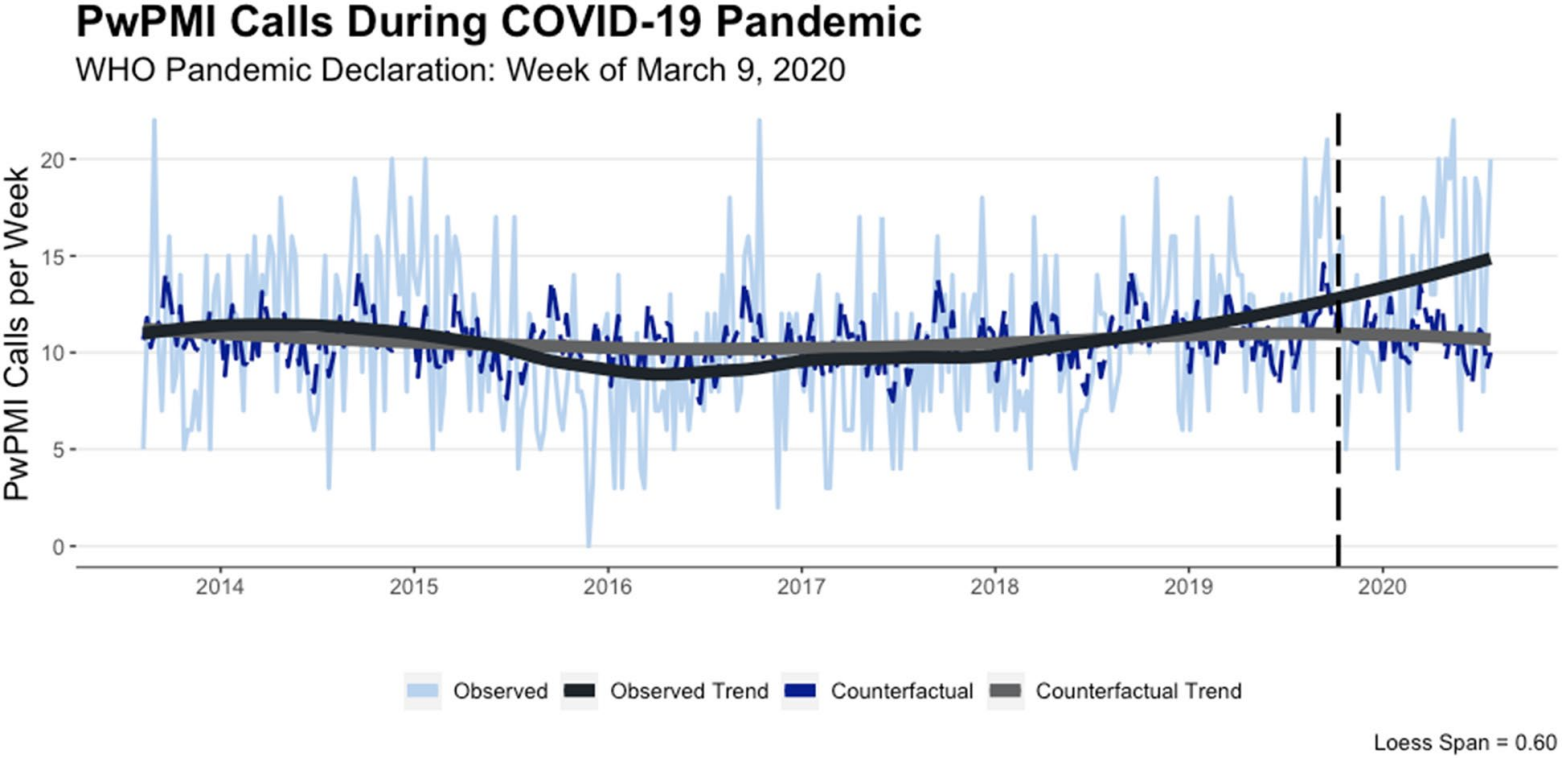
PwPMI Calls During COVID-19 Pandemic

**Fig. 2:**
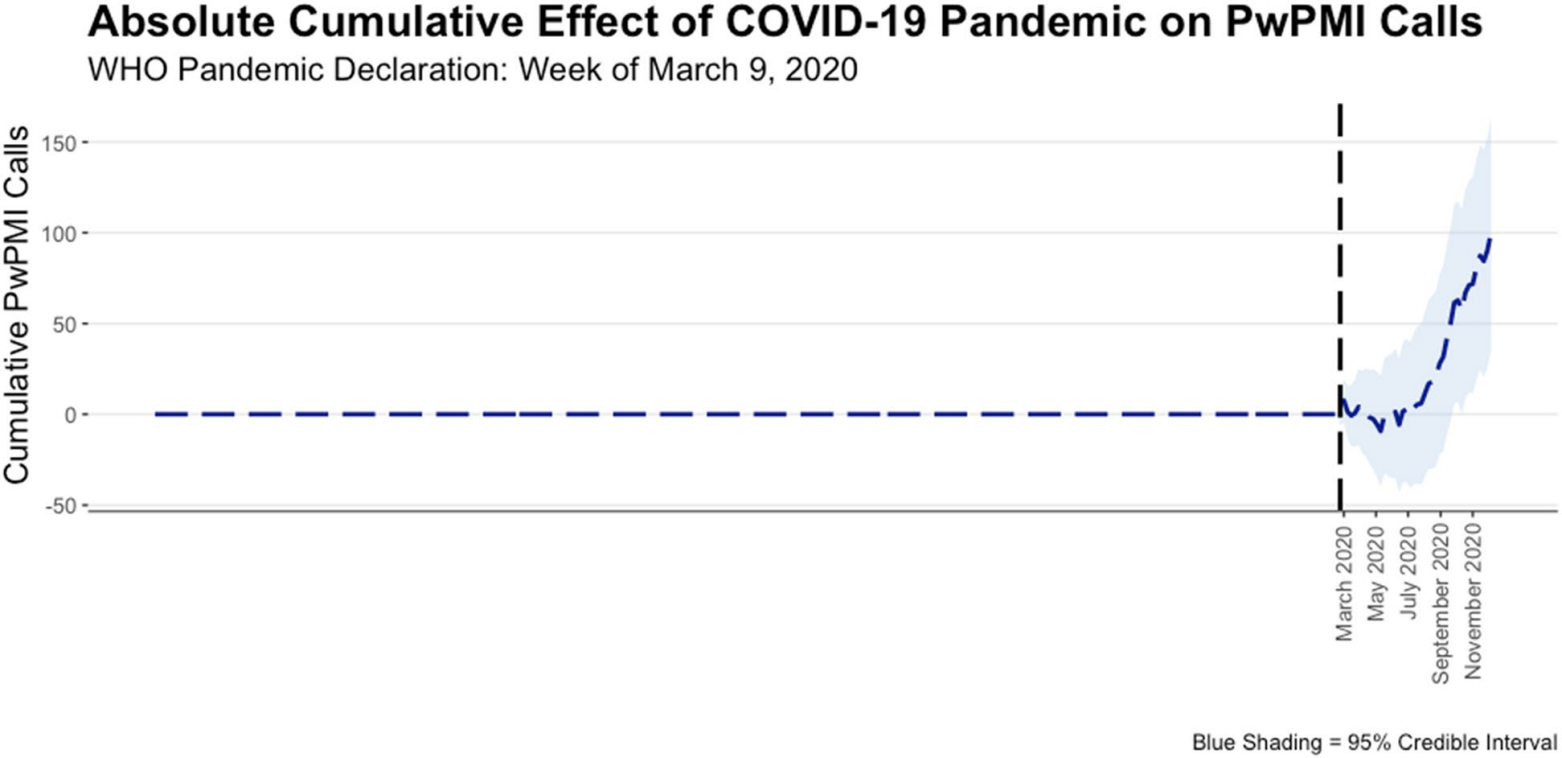
Absolute Cumulative Effect of COVID-19 Pandemic on PwPMI Calls

Figure 1 displays the observed and counterfactual time-series, along with their respective trends over time. Both time-series largely exhibited a similar weekly trend, but a divergence was noted immediately pre-intervention that is sustained and becomes more pronounced in the post-intervention period.^4^ A closer examination at the difference between the observed and counterfactual time-series post-intervention is displayed in Fig. 2. More specifically, in the weeks between March and July 2020, there appears to be no notable difference between the observed and counterfactual time-series. This, however, changes during the weeks of August 2020 where the difference between the time-series begins a sustained positive increase. Importantly, the 95% credible interval plotted in Fig. 2 includes zero until October 2020. As such, the difference between the observed and counterfactual time-series can only be considered as statistically significant in the weeks of October 2020 or later.

Table 2 presents the posterior inference for the BSTS model. The model estimated that there would be a mean of 11 PwPMI calls per week during the post-intervention period (*SD* = 0.78, CI = 9.4–12), however a mean of 13 was observed. In sum, during the entire 41-week post-intervention period, the Barrie Police Service received approximately 22% (*SD* = 7.1, CI = 8–36) more PwPMI calls than was predicted (*n* = 100.3, *SD* = 32.57, CI = 35.55–163.1). The posterior probability of the BSTS model is 99.9% (*p* < 0.001), suggesting that the COVID-19 pandemic was associated with a statistically significant increase in PwPMI calls for police service.

**Table 2:** Posterior Inference, Bayesian Structural Time-Series Model

	Average	Cumulative
Actual	13	557
Prediction (SD)	11 (0.78)	457 (32.57)
95% CI	[9.4, 12]	[393.9, 520]
Absolute Effect (SD)	2.4 (0.78)	100.3 (32.57)
95% CI	[0.87, 3.9]	[35.55, 163.1]
Relative Effect (SD)	22% (7.1%)	22% (7.1%)
95% CI	[8%, 36%]	[8%, 36%]

Bayesian One-Sided Tail-Area Probability: *p* < .001

Posterior Probability of a Causal Effect: 99.9%

## Conclusions

The purpose of the present study was to extend previous work into the examination on the effect of the COVID-19 pandemic on PwPMI calls for police service. While the only previous inquiry by Lersch (2020) found that PwPMI calls decreased in Detroit, Michigan, their study design was largely descriptive in nature and only drew upon data through April 27, 2020. As such, Lersch’s (2020) work was not optimally designed to detect the effect of an intervention, nor was the post-intervention time-series long enough to adequately measure the effect of the COVID-19 pandemic on PwPMI calls. The present study, therefore, sought to improve upon this work by employing a more robust methodological approach that drew upon a longer time-series.

The findings revealed that between March and July 2020, there was no difference between the observed number of PwPMI calls and what would have been expected had the COVID-19 pandemic not occurred. In August 2020, however, the observed number of PwPMI calls began a sustained positive increase away from what was predicted by the BSTS model. This difference between observed and expected time-series later became statistically significant in October 2020 and ultimately accounted for a 22% increase in PwPMI calls relative to what was expected. In other words, this study found that the COVID-19 pandemic has had an effect on PwPMI calls for police service, but that the effect was not immediate. While it is difficult to point to any particular pandemic-related factor, or combination of factors, that may have contributed to the observed increase in PwPMI calls, it is worth noting that the study jurisdiction entered into the second wave of the COVID-19 pandemic in September 2020 after low COVID-19 case-counts and easing of public health restrictions over the summer months (Nielsen, 2020). Longitudinal studies into the mental health effects of the COVID-19 pandemic have found that heightened levels of anxiety, stress, and depression reported at the onset of the pandemic may be sustained over time (Czeisler et al., 2021; Vahratian et al., 2021), thus it is possible that the emergence of the second wave may have exacerbated mental health problems spurred on by the first wave to the point of requiring police involvement.

From a practical perspective, while the role of the police as responders to mental health issues in the community has been a highly-debated topic even before the COVID-19 pandemic (Thompson, 2010; Wilson-Bates, 2008)—and especially since the defund the police movement (Koziarski & Huey, 2021)—it is clear this role has become more pronounced during the pandemic and thus will likely add to this debate. Fortunately, though, as the present study reveals, this more pronounced role did not begin until five months after the COVID-19 pandemic began. As such, this points to a need for policymakers to prioritize widely accessible mental health care that can be deployed early during public health emergencies. Doing so can possibly mitigate or eliminate the need for police involvement later on, as was the case here. If a response is required, however, and in-light of increasing calls for civilian-based responses (Watson et al., 2021), recent research warns us that calls for service which are initially reported as involving PwPMI may in fact encompass other components that would require a police response (Lum et al., 2021; Ratcliffe, 2021). Future work is therefore needed as to how the police can safely and appropriately triage PwPMI calls with their local mental health partners, and particularly so during public health emergencies which can lead to an increase in such calls. Additional research is also needed that replicates the present study in other jurisdictions, as is multivariate research that builds upon the univariate limitation of the current study. In doing so, a more thorough understanding around the effect the COVID-19 pandemic has had on PwPMI calls can be obtained.

## Supplementary Information

**Additional file 1.** Sensitivity analysis, posterior inference table, Bayesian Structural Time-Series Model.

## Data Availability

The data are not available as they are confidential.

## Footnotes

1 Mental health calls are often coded as such by responding officers due to a perception or belief that mental health-related issues *may* be present, as opposed to a *certainty* that they are present. In light of this, Frederick and colleagues (2018) recommend that empirical efforts which rely on officer-generated data should employ terminology that reflects this uncertainty.

2 ‘Suicide in Progress’, ‘Suicide Threats’, ‘Mentally Ill Person’.

3 The only study to temporally examine PwPMI calls concluded that “these variables [temperature and precipitation] cannot be expected to have a large magnitude impact on MHA [Mental Health Act] calls for police service.” (Vaughan et al., 2019, p. 181).

4 As is evident in Table 1 and Fig. 1, there was quite a substantial drop in PwPMI calls in 2016 that the counterfactual time-series was unable to account for. Given the univariate nature of the present BSTS modelling, a sensitivity analysis was conducted to examine the influence this drop in PwPMI calls may have had on the final results. For this sensitivity analysis, the entirety of 2016 was removed from the time-series and weekly call counts from 2014 and 2015 were shifted into the fields for 2015 and 2016, respectively, to fill the ‘gap’ in the series. A BSTS model was subsequently estimated using this modified data. Findings of the sensitivity analysis revealed that 464 (*SD* = 32.06, CI = 400.8–525) PwPMI calls were expected during the post-intervention period, whereas the model with 2016 included estimated 457 (*SD* = 32.57, CI = 393.9–520) PwPMI calls during the post-intervention period (see Table 2). This suggests that the drop in PwPMI calls in 2016, and the inability of the counterfactual time-series to detect it, has almost no impact on the final results. To see the posterior inference table for this sensitivity analysis, please see the online supplementary material (Additional file 1).
