# Supplementary material for: The effect of the COVID-19 pandemic on mental health calls for police service
Source: Crime Sci. 2021 Oct 11;10(1):22. doi: 10.1186/s40163-021-00157-6 (PMC8503731; doi:10.1186/s40163-021-00157-6)
Supplement: Supplementary file 1 — Additional file 1. Sensitivity analysis, posterior inference table, Bayesian Structural Time-Series Model. [file 40163_2021_157_MOESM1_ESM.docx]

**CRSC-D-21-00014.R1: Supplementary Document**

Sensitivity Analysis: Koziarski, J. (2021). The Effect of the COVID-19 Pandemic on Mental Health Calls for Police Service. *Crime Science*. Forthcoming.

As is evident in Table 1 and Figure 1, there was quite a substantial drop in PwPMI calls in 2016 that the counterfactual time-series was unable to account for. Given the univariate nature of the present BSTS modelling, a sensitivity analysis was conducted to examine the influence this drop in PwPMI calls may have had on the final results. For this sensitivity analysis, the entirety of 2016 was removed from the time-series and weekly call counts from 2014 and 2015 were shifted into the fields for 2015 and 2016, respectively, to fill the ‘gap’ in the series. A BSTS model was subsequently estimated using this modified data. Findings of the sensitivity analysis, presented below, revealed that 464 (*SD*=32.06, CI=400.8-525) PwPMI calls were expected during the post-intervention period, whereas the model with 2016 included estimated 457 (*SD*=32.57, CI=393.9-520) PwPMI calls during the post-intervention period (see Table 2 in text). This suggests that the drop in PwPMI calls in 2016, and the inability of the counterfactual time-series to detect it, has almost no impact on the final results.

|  | **Average** | **Cumulative** |
| --- | --- | --- |
| **Actual** | 13.00 | 557 |
| **Prediction (SD)** | 11 (0.76) | 464 (32.06) |
| **95% CI** | [9.5, 13] | [400.8, 525] |
|  |  |  |
| **Absolute Effect (SD)** | 2.2 (0.76) | 93.4 (32.06) |
| **95% CI** | [0.75, 3.7] | [31.60, 156.2] |
|  |  |  |
| **Relative Effect (SD)** | 20% (6.9%) | 20% (6.9%) |
| **95% CI** | [6.8%, 34%] | [6.8%, 34%] |
|  |  |  |
| Bayesian One-Sided Tail-Area Probability: *p* < .001 | | |
| Posterior Probability of a Causal Effect: 99.9% | | |
